# Study on the Regulatory Mechanism of oar-miR-29b in Lamb Encephalitis Caused by *Enterococcus faecalis* Infection

**DOI:** 10.3390/genes17010029

**Published:** 2025-12-29

**Authors:** Ming Zhou, Borui Qi, Pengfei Zhao, Longling Jiao, Shuzhu Cao, You Wu, Jingjing Ren, Runze Zhang, Yongjian Li, Yayin Qi

**Affiliations:** College of Animal Science & Technology, Shihezi University, Shihezi 832003, China; 18119829127@163.com (M.Z.); 18709646925@163.com (B.Q.); m18317351285@163.com (P.Z.); 13364947693@163.com (L.J.); wuyou1180@163.com (Y.W.); zrz2625874094@163.com (R.Z.); 13625425542@163.com (Y.L.)

**Keywords:** *Enterococcus faecalis*, inflammatory injury, microRNA, oar-miR-29b, tight junction

## Abstract

Background: *Enterococcus faecalis* is an opportunistic pathogen that is capable of causing bacterial encephalitis under specific pathological conditions. MicroRNAs (miRNAs) are a class of small, single-stranded non-coding RNAs, typically approximately 21 nucleotides in length. As master regulators of gene expression, they orchestrate critical pathways across diverse organisms and a broad spectrum of diseases; however, their role during *E. faecalis* neuro-invasion remains unexplored. Methods: A lamb model of *E. faecalis*-induced encephalitis was established. Integrated analysis of high-throughput sequencing data identified oar-miR-29b as a key differentially expressed miRNA during infection. To first verify its association with inflammation, primary SBMECs were stimulated with lipoteichoic acid (LTA), confirming that oar-miR-29b expression was significantly upregulated under inflammatory conditions. Subsequently, independent gain- and loss-of-function experiments in SBMECs were performed, with inflammatory cytokine expression assessed by qPCR and tight-junction protein levels evaluated by Western blotting. Results: Functional studies demonstrated that oar-miR-29b acts as a pro-inflammatory mediator, significantly upregulating IL-1β, IL-6, and TNF-α while degrading tight-junction proteins (ZO-1, occludin, and claudin-5), thereby compromising endothelial barrier integrity. Mechanistically, bioinformatic prediction and dual-luciferase reporter assays confirmed C1QTNF6 as a direct target of oar-miR-29b. The oar-miR-29b/C1QTNF6 axis is thus defined as a novel regulatory pathway contributing to neuro-inflammation and blood-brain barrier disruption. Conclusions: Collectively, our findings identify the oar-miR-29b/C1QTNF6 axis as a novel pathogenic mechanism that exacerbates *E. faecalis*-induced neuroinflammation and blood-brain barrier disruption.

## 1. Introduction

Enterococcus faecalis is a commensal bacterium in the digestive tracts of humans and animals. Certain strains are used in the medical field as probiotics to treat diarrhea [1,2]. Some strains also produce bacteriocins, which effectively inhibit bacterial growth and development in livestock production. They restore intestinal flora balance, enhance the body’s resistance, and improve livestock productivity [3]. However, *E. faecalis* itself is also a conditionally pathogenic bacterium, and its potential pathogenic risks should not be overlooked. In recent years, cases of endocarditis, meningitis, sepsis, and urinary tract infections in both humans and animals caused by mixed *E. faecalis* infections have also frequently occurred [4].

MicroRNAs (miRNAs) are predicted to regulate the activity of over 60% of protein-coding genes, participating in the regulation of nearly all cellular biological processes. They regulate protein synthesis by forming complementary base pairs with target mRNAs. In living organisms, miRNAs can form partial hybridization with the 3′ untranslated regions (UTRs) of mRNAs, thereby modulating their expression and consequently influencing the production of corresponding proteins. For example, miR-483 can act as a potent cancer promoter in one scenario while serving as a guardian of tissue stability in another [5]; miR-21 can suppress/promote IL-12 expression, playing distinctly opposite roles in different diseases [6,7]; in chronic lymphocytic leukemia (CLL), downregulated miR-15 and miR-16 suppress leukemic cell apoptosis and promote uncontrolled proliferation [8]; and miR-205-5p has been identified as a potential predictive biomarker distinguishing pancreatic cancer from chronic pancreatitis patients, with an accuracy rate of 86.7% [9]. Additionally, miRNAs such as miR-21 and miR-196a exhibit high discriminatory power in non-small cell lung cancer (NSCLC) patients [10].

In recent years, research findings on miRNAs in encephalitis have proliferated. For instance, studies revealed that extracellular miR-432-5p, miR-4433b-5p, and miR-599 correlate with the severity of anti-NMDAR encephalitis and may serve as potential biomarkers for disease monitoring [11]. MicroRNAs can influence inflammatory responses in bacterial encephalitis by targeting inflammation-related genes. For instance, miR-155-5p directly targets *SOCS5* to activate the JAK2/STAT3 pathway [12]. In blood–brain barrier studies, miRNAs regulate barrier integrity and influence the transmembrane transport of pathogens and immune cells. For instance, pericyte-derived exosomal miR-210 improves mitochondrial function and suppresses lipid peroxidation in vascular endothelial cells after traumatic spinal cord injury by activating the JAK1/STAT3 signaling pathway [13].

The miR-29 family (miR-29a/b/c) has evolved into a multi-organ, multi-disease, multi-mechanism network whose core functions converge on three overarching themes [14]: extracellular-matrix homeostasis, metabolic control, and immune regulation. For example, in terms of anti-fibrosis, overexpression of miR-29a/b significantly reduced hydroxyproline content and α-SMA positive area [15]; in terms of anti-inflammatory and immune regulation, research has found that that in human dendritic cells (DCs), the intracellular pattern recognition receptor NOD2 was activated and the expression of miR-29b was rapidly upregulated [16]. At the metabolic level, miR-29a mitigates myocardial ischemia/reperfusion injury in rats by activating the SIRT1/AMPK/PGC1α pathway [17]. miR-29b has emerged as a pleiotropic tumor suppressor; in mouse models it simultaneously downregulates multiple oncogenes—including *SP1*, *CDK6*, *DNMT3a*, and *DNMT3b*—to inhibit malignant growth [18].

Although miR-29b has been implicated in various non-infectious diseases, its role in bacterial infections—particularly *E. faecalis*-induced meningoencephalitis—remains unexplored. We observed a sustained upregulation of oar-miR-29b in lamb brain tissue at 48 and 72 h post-infection via RNA sequencing (|log_2_FC| > 1, FDR < 0.01). Given the dual (pro- or anti-inflammatory) activity reported for miR-29b [19,20], we hypothesized that it contributes to the pathogenesis of *E. faecalis*-induced meningoencephalitis. To test this hypothesis, we stimulated SBMECs with lipoteichoic acid (LTA) to simulate inflammation, which confirmed the upregulation of oar-miR-29b under inflammatory conditions. Subsequently, to define the regulatory role of oar-miR-29b, we performed gain- or loss-of-function experiments in SBMECs by overexpressing or inhibiting oar-miR-29b. qPCR and Western blot analyses revealed that oar-miR-29b upregulation significantly enhanced the expression of IL-1β, IL-6, and TNF-α, whereas its inhibition attenuated these cytokines. Concurrently, miR-29b overexpression markedly reduced the levels of tight-junction proteins (ZO-1, occludin, and claudin-5), indicating compromised blood–brain barrier integrity. Bioinformatic screening (TargetScan and RNAhybrid) and dual-luciferase reporter assays identified C1QTNF6 as a direct target of oar-miR-29b. Silencing C1QTNF6 recapitulated the pro-inflammatory and barrier-disrupting effects of miR-29b overexpression, whereas restoring its expression reversed these phenotypes. In summary, our findings demonstrate that oar-miR-29b promotes neuroinflammation and tight-junction disruption during *E. faecalis* infection by targeting C1QTNF6, revealing a novel miRNA-mediated mechanism in Gram-positive bacterial meningoencephalitis. This study aimed to determine whether oar-miR-29b exacerbates *E. faecalis*-induced lamb encephalitis by targeting C1QTNF6 and disrupting blood–brain barrier integrity.

## 2. Materials and Methods

### 2.1. Lamb Encephalitis Model Tissue Processing and RNA Extraction

The brain tissue of the lamb encephalitis model [21] constructed in the laboratory was cut into an appropriate size and placed in a non-enzymatic mortar. The grinding rod was used to pour liquid nitrogen while grinding to a fine dry powder, and then the total RNA was extracted by TRLZOL method.

### 2.2. Construction of Whole-Transcriptome Sequencing Libraries

After the quality of the total RNA samples was qualified, the RNA samples were sent to Beijing Nuohe Zhiyuan Technology Co., Ltd. (Beijing, China) for whole-genome transcriptome sequencing analysis to construct a library.

### 2.3. Target Gene Prediction and Functional Enrichment Analysis

To elucidate the potential functions of differentially expressed miRNAs, we predicted their target genes. Given that the study samples were sheep brain tissues (animal origin), we concurrently employed two animal-specific target prediction tools: miRanda (v3.3a) and RNAhybrid (v2.0). miRanda predicts based on sequence complementarity and thermodynamic stability, while RNAhybrid focuses on calculating minimum free energy (ΔG). Integrating the results from both tools (by taking their intersection) enhances prediction specificity and reduces false positives. The final high-confidence target gene set underwent Gene Ontology (GO) and Kyoto Encyclopedia of Genes and Genomes (KEGG) pathway enrichment analysis using the clusterProfiler R package (v3.8.1). This package provides statistical testing and multiple correction functions, with significant enrichment determined at *p*_adj_ < 0.05.

### 2.4. Cell Resuscitation and Culture

SBMECs were purchased from Mirror Image (Shanghai) Cell Technology Co., Ltd. (Shanghai, China). SBMECs were cultured in endothelial cell-specific medium supplemented with 15% bovine serum, 100 u/mL penicillin, 100 u/mL streptomycin, and endothelial growth factor in a 5% carbon dioxide incubator at 37 °C.

### 2.5. LTA- and LPS-Induced Inflammation in SBMECs

Primary SBMECs were passaged to the third generation and seeded into 6-well plates, cultured until reaching 70–80% confluence. To establish an inflammatory model, cells were stimulated for 6 h under standard culture conditions (37 °C, 5% CO_2_): the experimental group was treated with 200 ng/mL lipoteichoic acid (LTA, purchased from Sigma-Aldrich, St. Louis, MO, USA); the positive control group received 1 μg/mL lipopolysaccharide (LPS, purchased from Solarbio, Beijing, China); and the control group was treated with an equal volume of sterile PBS [22].

### 2.6. Total RNA Extraction and Fluorescence Quantification

The total RNA of the cells was extracted using the cell RNA rapid extraction kit, and the extracted RNA was subjected to concentration determination and normalization. Subsequently, the normalized RNA was synthesized into cDNA using a reverse transcription kit. For the determination of mi RNA expression, the stem-loop method was used to perform miRNA reverse transcription on the sample, and the stem-loop primers were designed using Novozymes primer design software (version 1.01). Sequence: (5′-GTCGTATCCAGTGCAGGGTCCGAGGTATTCGCACTGGATACGACACACTG-3′) q PCR. SYBR Green PCR premix was used for qPCR. U6 was used as the internal reference of miRNA, and β-actin was used as the internal reference primer sequence of mRNA, as shown in Table 1.

### 2.7. Cell Transfection

The short hairpin RNA of oar-miR-29b mimic/inhibitor and the corresponding negative control were purchased from GenePharma (Shanghai, China). The normally cultured SBMECs were washed with PBS 3 times and digested with trypsin. After the cells became rounded under the microscope, the trypsin was discarded, and the digestion was terminated with complete medium. The cells were scraped off with a scraper and resuspended. After fully being resuspended, the cells were counted and plated (3 × 104 cells/well) for 12 h, and then serum-free Opti-MEM (31985070 (Thermo Fisher Scientific, Waltham, MA, USA)) was added for starvation for 30 min. According to the manufacturer’s protocol, Lipofectamine 3000 reagent (Thermo Fisher Scientific) was used for transient transfection, and cells were harvested 24/48 h after transfection for downstream analysis (qPCR at 24 h and Western blot at 48 h post-transfection).

### 2.8. Western Blot Analysis

A 100 µL cell lysis buffer was added to the previously treated SBMECs. After 15 min of action, the cells were scraped off with a cell scraper and acted on the ice again for 15 min. After the cells were broken by an ultrasonic crusher, the cells were centrifuged at 12,000 r/s for 10 min at 4 °C using a low-temperature ultracentrifuge, and the supernatant was taken as the required protein. BSA was used to determine the protein concentration. According to the protein concentration, 10% and 6% sodium dodecyl sulfate-polyacrylamide gel electrophoresis were used. After electrophoresis, the protein was transferred to the nitrocellulose membrane and then blocked with 5% skimmed milk powder. Two hours later, for 10 min each time, TBST was used for cleaning 3 times. After that, the target primary antibody was added at 4 °C overnight. The membrane was incubated with secondary antibody at room temperature for 1 h. The chemiluminescence substrate of Western blot was the ECL kit, used according to the manufacturer’s instructions (Servicebio, Wuhan, China, Cat #G2161-200ML). The bands were analyzed using ImageJ 1.6 (National Institutes of Health, Bethesda, MD, USA) and normalized to anti-β-actin levels.

### 2.9. Dual-Luciferase Verification Report

The wild-type and mutant C1QTNF6 fragments containing the predicted oar-miR-29b binding site were constructed into the GP-mirGLO vector, respectively. The 293T cells were routinely cultured in DMEM medium containing 10% FBS (containing 1.5 mg/L glutamine, 100 U/mL penicillin, and 100 μg/mL streptomycin) at 37 °C, with a 5% CO_2_ saturated humidity incubator. When the cells were cultured to a density of 60%, according to (1) C1QTNF6 WT, NC; (2) C1QTNF6 WT, oar-miR-29b mimics; (3) C1QTNF6 MUT, NC; (4) C1QTNF6 MUT, oar-miR-29b mimics; (5) empty, NC; (6) EMPTY, oar-miR-29b mimics; (7) PC, NC; and (8) PC and oar-mir-29B mimics were co-transfected. After 48 h, according to the manufacturer’s specifications, the dual-luciferase reporter gene assay system (promega, Madison, WI, USA) was used to quantify the luciferase activity.

### 2.10. Data Analysis

Results GraphPad Prism 8 (GraphPad software) was used to process various test data, and one-way analysis of variance was used for comparison between groups. ns: not significant, *p* > 0.05. *: *p* < 0.05 indicates significant difference; **: *p* < 0.01 indicates that the difference is significant; ***: *p* < 0.001 indicates that the difference is extremely significant.

## 3. Results

### 3.1. Analysis of Transcriptome Sequencing Results

To investigate the impact of *E. faecalis* infection on sheep brain tissue, we performed transcriptome sequencing and identified differentially expressed miRNAs and mRNAs, followed by GO and KEGG pathway enrichment analyses.

**Figure 1:**
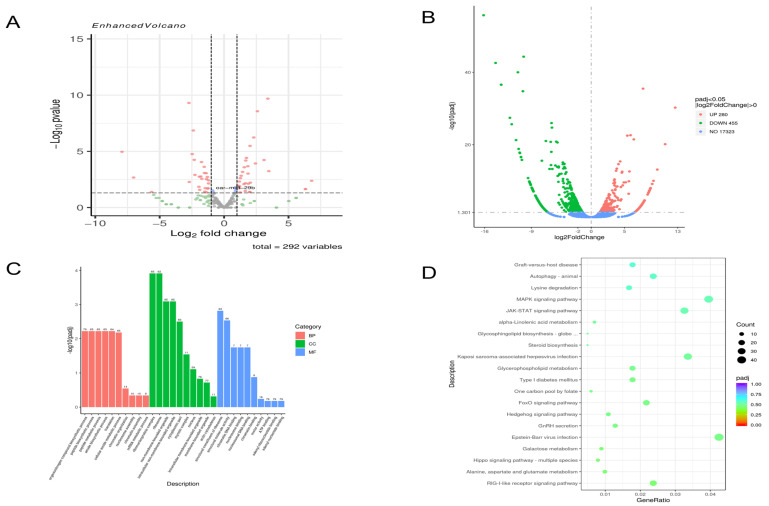
Analysis of 48 h transcriptome sequencing results (**A**). The 48 h miRNA volcano map. (**B**). The 48 h mRNA volcano map. (**C**). The 48 h GO enrichment analysis results histogram. (**D**). The 48 h KEGG enrichment results bubble diagram.

At 48 h post-infection, compared to the NC group, 115 miRNAs were upregulated, and 177 were downregulated (Figure 1A), while 280 mRNAs were upregulated, and 455 were downregulated (Figure 1B). GO enrichment analysis of these differentially expressed mRNAs revealed that in cellular components, the most significantly enriched terms were “intracellular non-membrane-bound organelle” and “intracellular membrane-bound organelle”. In biological processes, “organic nitrogen compound biosynthetic process” and “peptide biosynthetic process” were prominent. For molecular function, “structural molecule activity” and “chromatin DNA binding” were significantly enriched (Figure 1C). KEGG pathway analysis indicated enrichment in multiple signaling pathways, including the MAPK and JAK-STAT signaling pathways (Figure 1D).

**Figure 2:**
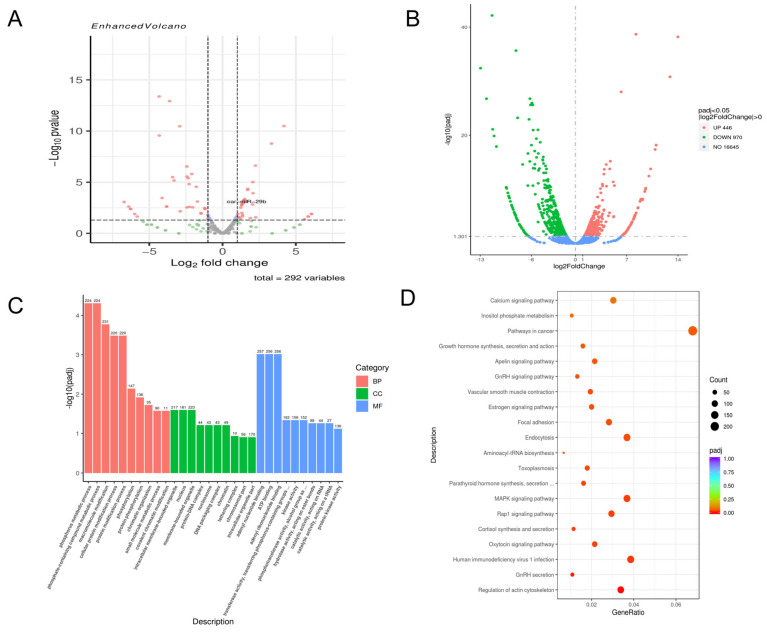
Analysis of 72 h transcriptome sequencing results (**A**). The 72 h miRNA volcano map. (**B**). The 72 h mRNA volcano plot. (**C**). The 72 h GO enrichment analysis results histogram. (**D**). The 72 h KEGG enrichment results bubble diagram.

At 72 h post-infection, 138 miRNAs were upregulated, and 154 were downregulated (Figure 2A), alongside 446 upregulated and 970 downregulated mRNAs (Figure 2B). GO analysis showed significant enrichment in metabolism-related biological processes, particularly phospholipid metabolism and cytochrome P450 metabolism. For cellular components, nucleus- and ribosome-related terms were highly enriched. Regarding molecular function, terms related to “structural molecule activity” and “ATP binding” were most significant (Figure 2C). KEGG analysis further showed enrichment in pathways such as the MAPK signaling pathway and various cancer-related pathways (Figure 2D).

Through the analysis of the sequencing results of the above model, we found that the expression of oar-miR-29b in the sequencing results was more special. It was upregulated at 48 h and 72 h, but the increase of 72 h was higher than that of 48 h. Because oar-miR-29b can play a pro-inflammatory or anti-inflammatory role by targeting different genes, we are not sure of its specific role in the lamb encephalitis model caused by *E. faecalis* infection. Therefore, we decided to construct a LTA treatment model of SBMECs for further exploration.

### 3.2. oar-miR-29b is Associated with Encephalitis

**Figure 3:**
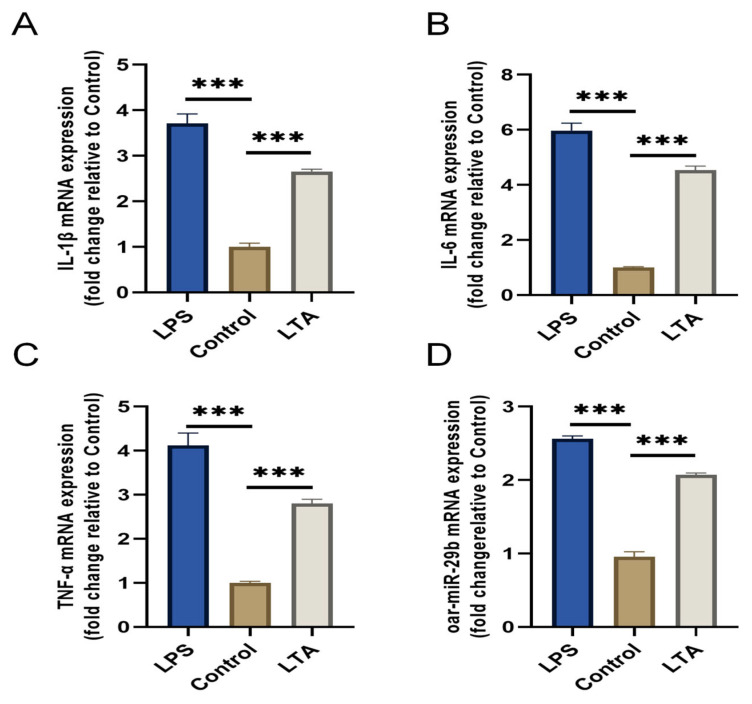
Expression of pro-inflammatory cytokines and oar-miR-29b in SBMECs following LPS or LTA stimulation. (**A**) IL-1β mRNA expression levels; (**B**) IL-6 mRNA expression levels; (**C**) TNF-α mRNA expression levels; (**D**) oar-miR-29b expression levels. Data are presented as mean ± SD of three independent experiments. *** *p* < 0.001 versus the untreated control group.

To investigate the response of oar-miR-29b to a Gram-positive inflammatory stimulus relevant to *E. faecalis* infection, we established an in vitro model using lipoteichoic acid (LTA) to stimulate primary SBMECs. LPS stimulation served as a positive control for general inflammation. Stimulation with LTA for 6 h successfully induced a pro-inflammatory state, as evidenced by the significant upregulation of IL-1β, IL-6, and TNF-α mRNA compared to the untreated control (Figure 3A–C). Notably, oar-miR-29b expression was also significantly upregulated specifically in response to LTA stimulation (Figure 3D). These results confirm that oar-miR-29b is dynamically upregulated in SBMECs under LTA-induced inflammation, supporting its potential involvement in Gram-positive bacterial neuroinflammation.

Through the above experiments, we preliminarily proved that oar-miR-29b can indeed be expressed in SBMECs, and that there is a correlation between oar-miR-29b expression and SBMECs under inflammatory treatment.

### 3.3. oar-miR-29b Can Regulate the Gene Expression of Inflammatory Factors in SBMECs and Is Related to the Integrity of the Tight-Junction Structure

**Figure 4:**
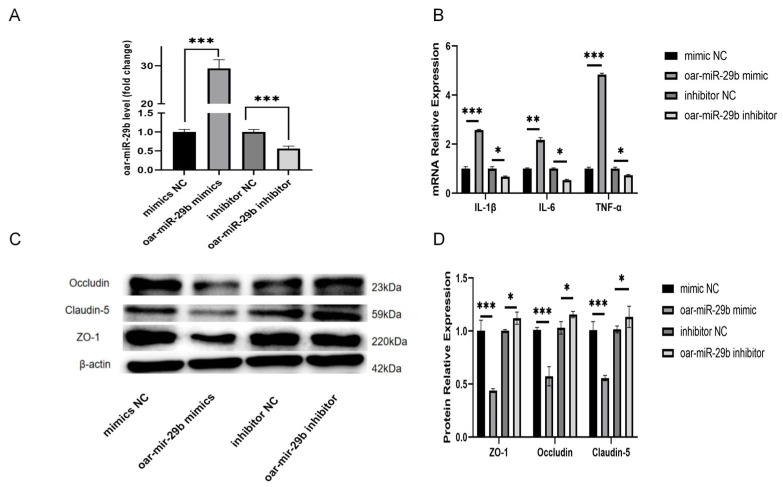
Oar-miR-29b regulates inflammatory response and tight-junction integrity in SBMECs; SBMECs were transfected with oar-miR-29b mimic (gain-of-function), its scrambled negative control (mimic NC), oar-miR-29b inhibitor (loss-of-function), or its scrambled negative control (inhibitor NC). (**A**) Experimental design for modulating oar-miR-29b function. SBMECs were transfected with oar-miR-29b mimic, inhibitor, or their respective negative controls (NC); (**B**) mRNA expression levels of the pro-inflammatory cytokines IL-1β, IL-6, and TNF-α; (**C**) Representative Western blot images showing protein levels of the tight-junction components ZO-1, occludin, and claudin-5; (**D**) Densitometric quantification of ZO-1, occludin, and claudin-5 protein levels from (**C**). Data in (**A**,**B**,**D**) are presented as mean ± SD of three independent experiments. Statistical significance was determined by one-way ANOVA followed by Tukey’s post-hoc test: * *p* < 0.05, ** *p* < 0.01, *** *p* < 0.001 indicate differences relative to the mimic NC/inhibitor NC group.

In order to study whether oar-miR-29b is indeed related to the inflammation of SBMECs, as detailed in Section 2.7, SBMECs were transfected with either oar-miR-29b mimic or oar-miR-29b inhibitor to achieve gain- or loss-of-function of oar-miR-29b, the expression of inflammatory factor mRNA was detected by q-PCR, and the expression of tight-junction proteins was detected by WB. The results showed that the mRNA levels of inflammatory factors TNF-α, IL-1β, and IL-6 were significantly increased when oar-miR-29b was overexpressed (Figure 4B). When oar-miR-29b was inhibited, the mRNA levels of inflammatory factors TNF-α, IL-1β, and IL-6 were significantly decreased (Figure 4B). At the same time, when oar-miR-29b was overexpressed, the expression levels of tight-junction proteins ZO-1, Occludin, and Claudin-5 were significantly reduced (Figure 4C,D). When oar-miR-29b was inhibited, ZO-1, Occludin, and Claudin-5 were significantly increased (Figure 4C,D). The results showed that oar-miR-29b could regulate the expression of inflammatory genes in SBMECs. When oar-miR-29b was overexpressed, the integrity of tight junctions was destroyed.

Through the above experiments, we verified that the expression of oar-miR-29b can regulate the expression of inflammatory-factor genes and tight-junction proteins in SBMECs, indicating that oar-miR-29b is related to lamb encephalitis. In order to study the changes of inflammatory factors and tight-junction proteins caused by oar-miR-29b by regulating which gene, we verified it by bioinformatics analysis, fluorescence quantification, and dual-luciferase assay.

### 3.4. Screening and Validation of oar-miR-29b Target Genes

**Figure 5:**
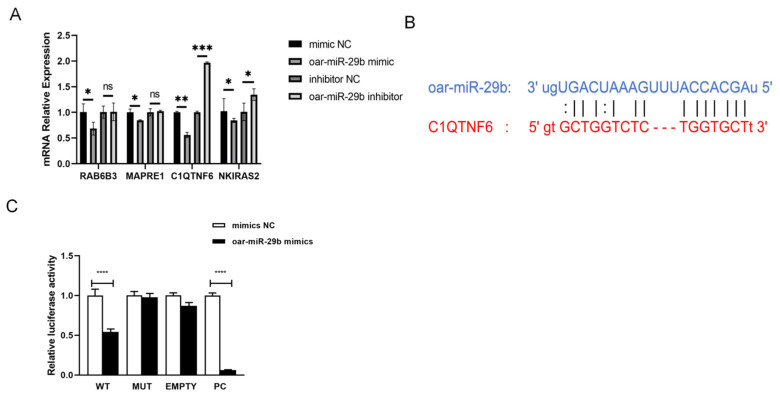
Identification of C1QTNF6 as a direct target of oar-miR-29b. (**A**) Validation of candidate target genes by qPCR. (**B**) Schematic of the predicted binding site of oar-miR-29b within the 3′-UTR of C1QTNF6. (**C**) Dual-luciferase reporter assay validating the direct interaction. Data in (**A**,**C**) are presented as mean ± SD of three independent experiments. Statistical significance was determined by one-way ANOVA followed by Tukey’s post-hoc test: * *p* < 0.05; ** *p* < 0.01; *** *p* < 0.001; **** *p* < 0.0001; ns, not significant (*p* > 0.05).

From the high-confidence candidate pool generated in Section 2.3, we finalized the candidate target by simultaneously considering (i) cross-species validation evidence (that is, genes previously identified as miR-29b targets in human or mouse systems) and (ii) the lowest BH-adjusted *p*-value (PADJ < 0.01) obtained from the enrichment analysis. The dual filter produced a focused subset (C1QTNF6, RAB6B3, NKIRAS2, and MAPRE1) for experimental verification. After overexpression and inhibition of oar-miR-29b in SBMECs, the expression changes of these target genes were observed to determine that there may be a certain correlation between C1QTNF6 and oar-miR-29b (Figure 5A). Subsequently, miRanda was used to predict the targets of mRNA and miRNA (Figure 5B). The predicted target was verified by double luciferase (Figure 5C). The results showed that oar-miR-29b had binding sites with C1QTNF6. The dual-luciferase verification report finally found that oar-miR-29 b had a certain specific binding ability with C1QTNF6. Collectively, these data from expression correlation, binding site prediction, and functional reporter assay confirm C1QTNF6 as a direct downstream target gene of oar-miR-29b in SBMECs.

## 4. Discussion

*E. faecalis* is a symbiotic bacterium that maintains the balance of intestinal microecology. However, when it breaks through the intestinal barrier and enters the blood circulation system, it may cause endocarditis. When it breaks through the blood–brain barrier, it may cause encephalitis, meningitis, and other diseases in diseased animals, and even lead to the death of diseased animals in severe cases [23]. MiRNA plays an important role in a variety of biological processes in recent years, including cell proliferation, differentiation, migration, and apoptosis. MiRNAs regulate gene expression by inhibiting the translation of specific mRNAs or directly cleaving them at the post-transcriptional level. In the central nervous system, miRNAs are involved in the regulation of mechanisms such as neurogenesis, neuroinflammation, oxidative stress, and apoptosis. Changes in their expression levels may drive the occurrence and development of neurodegenerative diseases such as AD through multiple pathways. At present, studies have shown that *E. faecalis* infection can lead to differences in the expression of some miRNAs in animal models, and these miRNAs can affect the role of *E. faecalis* on the host. For example, the supernatant of *E. faecalis* induces BMSC migration through the miR-200a-3p/FOXJ1/NFκB/MMPs axis [24]. Transcriptome profiling of lamb brain tissue revealed a sustained upregulation of oar-miR-29b at 48 h and 72 h post-infection, with levels at 72 h significantly exceeding those at 48 h (|log_2_FC| > 1, FDR < 0.01). Consequently, oar-miR-29b was selected for functional characterization.

miR-29b has been ascribed both pro- and anti-inflammatory roles. In dendritic cells, miR-29b overexpression shifts the phenotype toward an anti-inflammatory state by suppressing the NF-κB, STAT3, MAPK, and JUN signaling pathways [19]. Conversely, in other contexts, it has been reported to amplify MAPK-dependent inflammation and oxidative stress by targeting SPPY1 [25]. Given this context-dependent functionality, we aimed to define the role of oar-miR-29b in Gram-positive neuroinflammation. Primary SBMECs were stimulated with lipoteichoic acid (LTA). Unexpectedly, oar-miR-29b expression was significantly upregulated following LTA stimulation. This confirmed its dynamic association with LTA-driven inflammation and prompted further investigation into its mechanistic role.

To dissect the cell-autonomous function of oar-miR-29b, SBMECs were transfected with oar-miR-29b mimics, inhibitors, or corresponding negative controls (NCs). Inflammatory responses were assessed 24 h post-transfection by quantifying IL-1β, IL-6, and TNF-α transcripts via qPCR. Barrier integrity was evaluated 48 h post-transfection by analyzing the protein levels of the tight-junction components ZO-1, occludin, and claudin-5 using Western blot. Gain-of-function of oar-miR-29b significantly increased pro-inflammatory cytokine expression and concurrently reduced the levels of all three tight-junction proteins. Conversely, its silencing produced the opposite effects. These results demonstrate that oar-miR-29b concurrently regulates both inflammatory output and microvascular tight-junction stability in the ovine cerebral endothelium.

MicroRNAs (miRNAs) bind to complementary sequences in the 3′-untranslated regions (3′-UTRs) of target messenger RNAs (mRNAs). This interaction typically triggers mRNA degradation or translational repression, thereby reshaping downstream gene expression networks. In human airway epithelia, C1QTNF6 has been shown to dampen particulate matter (PM)-induced inflammation by inhibiting the AKT/NF-κB signaling axis, thereby reducing the generation of IL-1β, IL-6, IL-8, and reactive oxygen species (ROS) [26]. Conversely, silencing of C1QTNF6 amplifies these inflammatory responses. Furthermore, Wang et al. [20] demonstrated that miR-29b directly targets and inhibits C1QTNF6, consequently exacerbating the production of these same cytokines and the oxidative burst in human bronchial epithelial cells (HBECs). To identify the effector genes mediating oar-miR-29b’s function in the ovine cerebral endothelium, we employed an integrated approach combining bioinformatic prediction (using TargetScan and RNAhybrid) with qPCR-based expression screening in SBMECs. Among the predicted targets, C1QTNF6 emerged as a leading candidate, showing a strong inverse correlation with oar-miR-29b expression levels in SBMECs. Dual-luciferase reporter assays confirmed that oar-miR-29b directly binds to a specific site (nucleotides 1587–1601) within the 3′-UTR of the ovine C1QTNF6 transcript and functionally represses its expression. Consistent with this regulatory mechanism, overexpression of oar-miR-29b in SBMECs led to increased expression of IL-1β, IL-6, and TNF-α, concurrently with a decrease in the protein levels of the tight-junction components ZO-1, occludin, and claudin-5. Taken together, our data support a model in which oar-miR-29b promotes neuro-inflammation and compromises blood–brain barrier (BBB) integrity in the ovine cerebral endothelium, at least in part, through the targeted downregulation of C1QTNF6.

This study has several limitations. First, the work was conducted using an in vitro monolayer system, which may not fully recapitulate the complexity of the intact neurovascular unit. Future studies involving in vivo rescue experiments, as well as the generation of C1QTNF6 knock-in or knock-out models, will be essential to formally establish causality. Second, it remains to be determined whether additional direct targets of oar-miR-29b or parallel signaling pathways also contribute to the observed effects on neuro-inflammation and BBB disruption.

## 5. Conclusions

Integrated bioinformatic analysis and expression profiling in a lamb encephalitis model initially identified oar-miR-29b as a key regulator associated with neuro-inflammation. This study further demonstrates that in ovine brain microvascular endothelial cells (SBMECs), oar-miR-29b directly targets and suppresses C1QTNF6 by binding to its 3′-untranslated region (3′-UTR). Functionally, oar-miR-29b overexpression elevates pro-inflammatory cytokines (IL-1β, IL-6, and TNF-α) while reducing key tight-junction proteins (ZO-1, occludin, and claudin-5), thereby compromising blood–brain barrier (BBB) integrity. Conversely, inhibition of oar-miR-29b restores C1QTNF6 expression, attenuates inflammation, and preserves barrier function. Collectively, these results delineate a novel pathogenic axis, oar-miR-29b/C1QTNF6, which exacerbates neuro-inflammation and BBB disruption, contributing to the pathogenesis of *E. faecalis*-induced encephalitis.

## Figures and Tables

**Table 1:** Primers for genes.

Gene	Primer Sequence (5′ to 3′)
*IL-1β*	AGCCGAGAAGTGGTGTTCTG
	TGGCCACCTCTAAAACGTCC
*IL-6*	GACACCACCCCAAGCAGACTA
	TGCCAGTGTCTCCTTGCTGTT
*TNF-α*	AACAGGCCTCTGGTTCAGACA
	CCATGAGGGCATTGGCATAC
*β-actin*	CCTCACTGCTTCCTTCTCTCTC
	CCTAGAAGCATTTGCGGTGG
*C1QTNF6*	TTTCGGTAAGCACCCAGTCC
	GTGGGTAATGAAGGGCCACA
oar-miR-29b	CGCGTAGCACCATTTGAAAT
	AGTGCAGGGTCCGAGGTATT
*RAB6B3*	CAGAGGAGGACACAAGCCAC
	ACAGAGCATAGACGGTTGCC
*MAPRE1*	CACAGCCGACTCAGTTCCTT
	AAGGAAGAAGCATGGCTACCG
*NKIRAS2*	CACTGACGGCTATGTCCTGG
	TTTCCCCCAACCCAACTTCC
U6	GCTTCGGCAGCACATATACT
	TTCACGAATTTGCGTGTCAT

## Data Availability

The datasets presented in this article are not readily available because the data are part of an ongoing study. Requests to access the datasets should be directed to qiyayin@shzu.edu.cn.
